# Reconciling Mystical Experiences with Naturalistic Psychedelic Science: Reply to Sanders and Zijlmans

**DOI:** 10.1021/acsptsci.1c00137

**Published:** 2021-06-08

**Authors:** Jussi Jylkkä

**Affiliations:** Department of Psychology, Åbo Akademi University, Biskopsgatan 2, 20500 Åbo, Finland

## Abstract

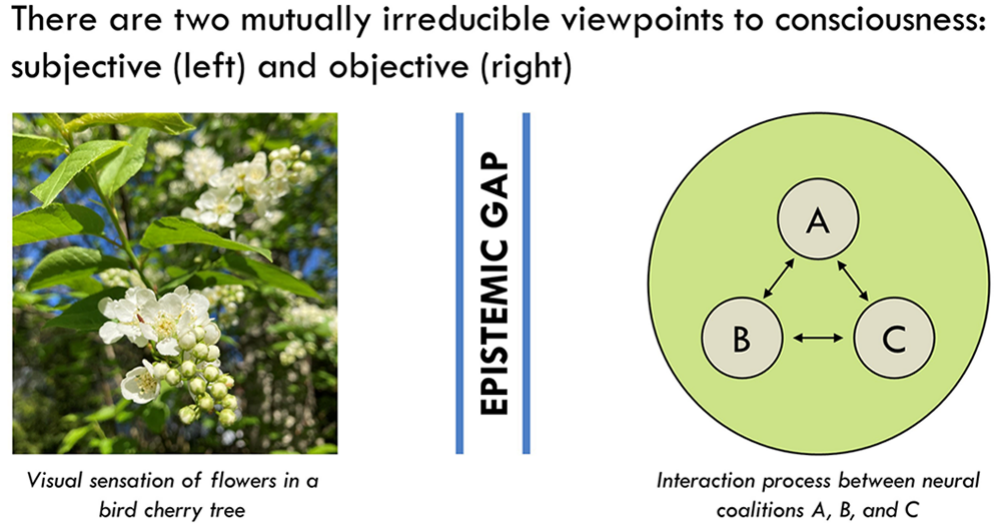

In a recent Viewpoint, Sanders and Zijlmans call for the demystification of psychedelic science. However, they ignore the subjective aspect of psychedelic experiences. For the subject, mystical experiences are felt as real and can yield personally meaningful insights. It is a philosophical question whether they are true.

In a recent Viewpoint article, Sanders and Zijlmans^1^ call for the demystification of the psychedelic experience. They argue that “there is an elephant in the room of modern psychedelic science”, namely, the mystical experience, which has been shown to mediate the therapeutic effect of psychedelic-assisted therapy. Sanders and Zijlmans argue that the notion of mystical experience conflicts with cognitive neuroscience or naturalism (*i.e.*, the ontological thesis that everything is physical, or the methodological thesis that science should stick to empirical methods^2^). For instance, the Mystical Experience Questionnaire (MEQ-30),^3^ commonly used in psychedelic research, includes questions about transcendence of space and time, meeting the ultimate nature of reality, experience of unity or that “all is one”, sense of sacredness or holiness, and ineffability. According to Sanders and Zijlmans, it is problematic for scientists to refer to such phenomena, which could be considered as supernatural.

Sanders and Zijlmans rightly raise the concern that the use of questionnaires like MEQ-30 can bias how the subject interprets their psychedelic experience. This is both a methodological and an ethical problem. First, it biases the empirical data, given evidence that the psychedelic experience is very sensitive to the so-called “set and setting”, *i.e.*, the psychological mindset of the subject and the physical and social context of the experience.^4^ Second, psychedelic experiences are highly meaningful and significant to the subjects. Thus, we should carefully respect their autonomy in interpreting their experiences.^5^ Of particular concern are subjects who identify themselves as atheists or nonspiritual. For such participants, more secular or neutral ways to assess the experience are needed.

However, not all mystical phenomena conflict with naturalism and science. Psychedelic experiences and consciousness in general are exceptional topics in science because subjective consciousness can never be studied completely objectively. From the subjective perspective, the psychedelic experience can indeed be ineffable and mystical and include metaphysical insights, but the truth of the insights is an independent philosophical question. For example, from the experience that all is one it does not necessarily follow that all is indeed one. Again, from the purely objective, neuroscientific perspective we cannot know the subjective aspects of experiences, but this does not imply that they would not exist or that the metaphysical insights would not be real for the subject.

I want to point to a future direction in psychedelic research where we would respect the metaphysical insights from a psychedelic experience for what they are, namely, philosophical intuitions which can be true or false. We should not reduce people’s worldviews to neuroscience or psychologize them but instead take as our premise that humans are rational agents who strive to believe what is true. I illustrate this approach by briefly presenting how insights of unity in psychedelic experience can be given a rational conceptualization that is compatible with the scientific worldview.

## The Subjective and Objective Viewpoints

Sanders and Zijlmans criticize the idea that psychedelic experiences are mystical and beyond the scope of empirical science. They hold that “current researchers should be optimistic at their prospects of creating valid frameworks that are supported by, and accessible to, empirical methods”. However, subjective experiences are exceptional as topics of scientific research. A large body of philosophical research demonstrates an *epistemic gap* between subjective experiences and science. For example, even if we knew everything about bats and their neurophysiology, we could not infer what it is like to be a bat.^6^ Likewise, a person who has never experienced colors can know all of neuroscience without knowing what colors look like.^7^ From purely empirical premises alone we cannot infer that subjective consciousness exists in the first place.^8^ The epistemic gap is denied only by a minority of philosophers, namely, eliminativists and illusionists, who deny the existence of subjective consciousness altogether or consider it as an illusion. Few would want to take psychedelic science into that extreme direction.

Ineffability is a characteristic of the mystical experience, but the epistemic gap demonstrates that all experience is ineffable. No one can describe the sound of a trumpet to a deaf person; knowing what an experience is like requires being able to experience it subjectively. However, this need not conflict with naturalism, as there are many naturalistic and materialistic strategies to explain what makes experiences ineffable.^9^ Of particular interest are Kantian approaches, which take the epistemic gap to demonstrate that our knowledge of matter is somehow limited.^10^ Importantly, this does not imply that consciousness would not be physical or that it could not be modeled scientifically.^11^ Science can in fact model consciousness, but the theoretical model should not be conflated with the concrete thing or process that the model is about, namely, consciousness itself. In short, the ineffability of consciousness does not necessarily conflict with natural science.

The epistemic gap shows that there are two mutually irreducible viewpoints to experience, subjective and objective. The subjects who undergo the psychedelic experience may from their own perspective consider that they have met the ultimate nature of reality, whereas from the objective perspective such mystical or metaphysical notions cannot be used. The neuroscientists, in turn, must limit their focus to what can be observed: what the subject reports of their experiences and what biochemical processes correspond to them. The neuroscientist can model the experiences that the subject describes as mystical, but the neuroscientist should not take a stand on whether the experiences are veridical or not. The veracity of the experience is an independent, philosophical question.

## The Importance of Philosophical Insights

Sanders and Zijlmans refer to the “ontological shock” related to psychedelic experience, a radical shift in the subject’s worldview. The experience may include philosophical and metaphysical insights which are radically new for the subject. For example, William James reported how he understood Hegel’s philosophy under the influence of nitrous oxide, and mescaline experience was a catalyst for Aldous Huxley’s “reducing valve” theory of consciousness. Reference to philosophical intuitions also play a key role in questionnaires like MEQ-30, represented by items such as “gain of insightful knowledge experienced at an intuitive level”, or experience of acquiring knowledge of the “ultimate nature of reality”.^3^

The psychedelic insights have what William James called “noetic quality” and are felt as true. It would not do justice to them to completely psychologize them or to treat them as merely neural processes. They are not just any kind of neural–psychological processes, but instead they form the subject’s worldview. To compare, also the naturalistic–materialistic worldview can be considered as a psychological process or a brain process, but its adherents consider it as depicting the world as it really is. To illustrate what kinds of philosophical insights psychedelic experience could afford, and how they could be rational and in line with the scientific worldview, I briefly discuss unitary experiences and panpsychist intuitions about the ultimate nature of reality.

Psychedelic experience can yield the unitary insight that “all is one”. From a philosophical perspective, this may be taken to represent monism, *i.e.*, the claim that everything belongs to one single ontological class. This claim need not conflict with naturalism or physicalism, because even physicalism itself is a monistic theory. It holds that everything, including consciousness, is physical. According to physics, the universe (from *unus*, the Latin word for one) is a unitary whole, and we are all forms of the same energy that originated in the Big Bang. This is compatible with psychedelic experiences of unity, if they are interpreted as the claim that everything belongs to one fundamental kind that constitutes reality.

What if the psychedelic subject claims to have gained insight into the ultimate nature reality? To illustrate, consider that the subject has come to believe that we are “waves in a sea of consciousness”. This could be elaborated as the thesis that consciousness is fundamental, or that everything that exists is continuous with consciousness. In philosophy, this is known as panpsychism. It would be ignorant to dismiss insights like this as contradictory with science, as panpsychism can be formulated in a way that is compatible with natural science and materialism.^10^ For example, it can be held that experiential properties ground the dispositions and relations that science describes, or that consciousness is part of the concrete reality that science models based on observations.^11^ Even if we are skeptical about such theories, we must admit that the compatibility between panpsychism and naturalism is an open philosophical question.

Mystical experiences may emphasize our ignorance of reality. It does not conflict with natural science to acknowledge that science is limited to modeling reality or that it cannot tell anything of the reality beyond observations and models. The physicist Stephen Hawking notes that science is “just a set of rules and equations” and continues to ask: “What is it that breathes fire into the equations and makes a universe for them to describe?”.^12^ This opens room for positive claims about the reality that transcends the scientific observations and models, such as panpsychism.

## Conclusion

I agree with Sanders and Zijlmans that psychedelic scientists should develop more neutral psychometric instruments to probe psychedelic experiences. However, we should not ignore the subjective aspects of psychedelic experiences and the metaphysical or even mystical insights associated with them. I have argued that we should not psychologize these intuitions or treat them as mere brain processes, because they constitute the subject’s worldview. It is a philosophical question whether they are true or compatible with the scientific worldview.
